# M. Bourgeois' Case of Scirrhus of the Stomach

**Published:** 1826-07

**Authors:** 


### DISEASE OF THE STOMACH.

In another part of this Journal we have given some interesting observations and cases of gastralgia by M. Barras, shewing how nervous affections of the stomach may sometimes be mistaken for inflammatory or organic diseases of that viscus, to the great detriment of the patient. The case which we are about to relate will set, in a striking point of view, the difficulties with which the medical practitioner is beset; and how liable he is, in avoiding Scylla, to run into Charybdis. Of the two errors, it is certainly better to mistake gastralgia for gastritis than the reverse. In the *former* case, the penalty is generally only a protraction of the cure, and a needless quantum of suffering—in the *latter*, the consequences may be fatal. It is only by a careful record and observation of facts that we can hope to avoid both errors.

*Case by M. Bourgeois.* (Journal General, January 1826.) In the beginning of the year 1817, a gentleman, (M. P.) aged 45, of high complexion, and inclining to corpulency, consulted our author for a slight gastralgia, which was generally felt after eating, accompanied by a sense of fulness, eructations, discharges of wind, and also a considerable flow of clear saliva, especially in the mornings. He also complained of pain over the orbits, loss of appetite, bitter taste in the mouth. The tongue was coated—and there was a yellowish tint, very slight, about the alæ nasi and commissures of the lips. A similar train of symptoms, the patient stated, had occurred several times during the preceding years —at first very slight, and fugitive—afterwards more troublesome, and of longer duration. He traced the commencement and the cause of the complaint, to the period of trouble when the Allies entered France, at which time he suffered great anxiety of mind. These attacks had generally given way to emetics, and the same remedy was at this time employed, but with only a very temporary good effect. M. Bourgeois, therefore, recommended abstinence, milk diet, leeches to the epigastrium and anus. These means procured a gradual and slow amelioration of the symptoms, and the patient was able to attend to his affairs. But the least error in regimen renewed all his sufferings, and the patient began to tire of the plan of rigid diet and lowering means recommended by his physician. A consultation was called, and the new physician looked upon the disease as gastralgic or nervous, and, therefore, reversed the treatment, giving opiates, bitters, rhubarb and magnesia, and the waters of Vichy. These means were continued for some weeks, but evidently with an aggravation of the complaint. The patient now began sensibly to emaciate, and he complained greatly of a heavy gnawing pain between the shoulder blades. The epigastrium was now tender on pressure—the bowels constipated, and there were distressing colicky pains. The patient was completely hypochondriacal, and excessively irritable.

The new plan of treatment was, in its turn, abandoned, and the patient determined to throw himself into the hands of empirics. Our author was, of course, dismissed, and did not meet with the patient for six months afterwards. The meeting then was accidental. M. Bourgeois perceived that the emaciation had considerably advanced—that the complexion was more plombeau—the eyes hollower—the features much more altered. Still, the patient was not laid up, and he evidently dissembled his real feelings, partly, perhaps, from shame at being in the hands of quacks. A time, however, arrived, when the unhappy patient could no longer disguise his situation. In the beginning of January, 1818, he again sent for M. Bourgeois, in the middle of the night; and was found by him, pale, emaciated, scarcely able to make himself understood, and apparently ready to be suffocated. His countenance expressed the greatest fear and anxiety—the pulse was small and intermitting. He took M. Bourgeois's hand, and carried it to the epigastrium, where, to the physician's astonishment, there was a solid tumour the size of a child's head, pulsating synchronously with the heart! While examining this tumour, a paroxysm of vomiting came on, and some slimy mucus was thrown up. This sickness, he was informed, took place whenever any thing was received into the stomach, whether liquid or solid. In short, it was abundantly evident that there was a scirrhus of the stomach, of a fatal kind. It is, therefore, needless to dwell on the means that were used—two only of which ever gave even temporary relief. These were leeches to the epigastrium, and laudanum thrown up by injections. Emaciation was at length carried to the utmost degree, and almost every part of the body was sympathetically affected by this dreadful malady. There was constant dry cough, and the mental functions were greatly disturbed. Nature, assisted by art, withstood this terrible trial for 47 days after M. Bourgeois's second summons to this unfortunate gentleman, when death put an end to his sufferings and miserable existence without a groan or a struggle.

*Dissection.* When the abdominal cavity was laid open, the tumour, which was easily recognized to be the stomach, was found immoveable, in consequence of intimate adhesions with all the neighbouring parts. It was carefully isolated and removed from the body. It was the size of a large melon—hard—and weighed five pounds French. The scalpel could hardly penetrate it. It was of a fibro-lardaceous nature, and quite homogeneous in appearance. It occupied, or rather consisted of, the coats of the stomach thus changed in structure. The cavity of the organ was very small, and the lining membrane was every where pale, or streaked with granulations. There was about an eighth part of the viscus, towards the superior portion, which was not involved in the scirrhous disorganization. The pyloric orifice was entirely lost in the mass of disease, and could not be recognized. The greater part of the duodenum, and the whole of the pancreas were involved in the ruin. There was nothing worthy of remark observable in any other part of the body. The thoracic organs were perfectly sound, though their functions had been so much disturbed by the gastric disease.

M. Bourgeois makes many sensible observations on this deplorable malady, which, he thinks, is much more common in our days than formerly. This is not improbable, on many accounts, but chiefly in consequense of the progress of luxury, and the increased cultivation of the mind at the expence of the body. The case which has been detailed, combines in itself most of the prominent or essential characters of this fatal disease. Among these, the slow and insidious march of the malady is the most conspicuous. A slight derangement of function in the stomach, with a trifling malaise, rather than pain, marked by a sense of fulness, weight, and constriction, principally during digestion, were prelusive of tongue furred in the middle and red at the edges and point—inappetence—disposition to nausea—acid or nidorous eructations—distressing flatulence. Such are the primitive symptoms; and it must be confessed that these are common to many affections of the stomach which have nothing of a malignant, nor even of an inflammatory nature about them—such as the common cases of indigestion or loss of tone in the stomach, from sedentary occupations, literary studies, anxieties of mind, and a hundred other causes.

According to our author's experience, the least equivocal symptoms of the serious malady now under consideration, are darting or lancinating pains—vomiting—a voracious craving for food (which he regards as a mode of pain peculiar to the stomach)—and a sensation of faintness. The last-mentioned symptom is an indication that the vital force is concentrated towards the disordered organ. Epigastric pulsation is also considered as a bad sign—but every man of experience must have seen this in numerous cases where no serious malady was in existence at the time.—We consider these pulsations as very far from being "les signes precurseurs de la tumeur pathognomonique."— He thinks, and with probability, that when the posterior portion of the stomach becomes first the seat of disease, it eludes investigation for some time, on account of the depth at which it is situated. In many cases, however, the symptoms of gastric scirrhus are so uncertain, that the disease arrives at a monstrous, or even fatal, height, without being recognized by the medical attendants; witness the memorable case of Napoleon Buonaparte. Still, we think an attentive observation of the phenomena, and especially of the effects of remedies, and the juvantia and lædentia, will enable us, in most instances, to distinguish cases of gastritis from those of mere gastralgia. We refer to the paper on the latter subject for illustration. In respect to the case just now related, we are very far from agreeing with our author, that the disease was owing merely to chronic inflammation of the stomach. It was clearly a specific disease, in all probability, inherent in the constitution, and which no system of regimen could have prevented—no plan of treatment cured. We do not, by this, mean to assert that a certain treatment (for instance, of stimulation) might not aggravate a scirrhous affection of the stomach or any other part, since irritation and inflammation must be ingredients in the morbid process going forward in this terrible disease. What we mean to say is this—that the stimulating treatment was not the cause of this patient's death—nor would all the leeches in Paris have saved his life, though they might probably have prolonged it.

M. Bourgeois, in adverting to the causes which have increased the prevalence of this disease in our days, accuses, among other things, the immoderate use of coffee—"whose *irritating* action on the sensorium is so marked, that it has obtained the title of *intellectual drink.*" We should be disinclined to accuse coffee of such a crime as that of producing cancer of the stomach—neither are we convinced of its *irritating* action on the sensorium, although, like strong tea, it has the effect of preventing sleep in most individuals. We do not conceive that irritation and *excitation* are perfectly synonymous in a physiological sense. The mildest food *excites* the stomach, and through that several other organs in the body—but are we justified in saying it *irritates* the stomach? The same may be said of coffee.

